# *DNMT3A* R882 Mutations Confer Unique Clinicopathologic Features in MDS Including a High Risk of AML Transformation

**DOI:** 10.3389/fonc.2022.849376

**Published:** 2022-02-28

**Authors:** Majd Jawad, Michelle Afkhami, Yi Ding, Xiaohui Zhang, Peng Li, Kim Young, Mina Luqing Xu, Wei Cui, Yiqing Zhao, Stephanie Halene, Aref Al-Kali, David Viswanatha, Dong Chen, Rong He, Gang Zheng

**Affiliations:** ^1^Division of Hematopathology, Department of Laboratory Medicine and Pathology, Mayo Clinic, Rochester, MN, United States; ^2^Division of Molecular Pathology and Therapy Biomarkers, Department of Pathology, City of Hope Comprehensive Cancer Center, Duarte, CA, United States; ^3^Division of Hematopathology, Department of Pathology, City of Hope Comprehensive Cancer Center, Duarte, CA, United States; ^4^Department of Laboratory Medicine, Geisinger Health, Danville, PA, United States; ^5^Department of Pathology, H. Lee Moffitt Cancer Center and Research Institute, Tampa, FL, United States; ^6^Department of Pathology, Associated Regional and University Pathologists (ARUP) Laboratories, Salt Lake City, UT, United States; ^7^Department of Pathology, Yale School of Medicine, New Haven, CT, United States; ^8^Department of Pathology & Laboratory Medicine, The University of Kansas Medical Center, Kansas City, KS, United States; ^9^Department of Preventive Medicine, Northwestern University, Chicago, IL, United States; ^10^Department of Internal Medicine, Division of Hematology, Yale School of Medicine, New Haven, CT, United States; ^11^Division of Hematology, Mayo Clinic, Rochester, MN, United States; ^12^Division of Laboratory Genetics and Genomics, Mayo Clinic, Rochester, MN, United States

**Keywords:** *DNMT3A*, genetics, R882 mutations, myelodysplastic syndromes, acute myeloid leukemia

## Abstract

*DNMT3A* mutations play a prominent role in clonal hematopoiesis and myeloid neoplasms with arginine (R)882 as a hotspot, however the clinical implications of R882 vs. non-R882 mutations in myeloid neoplasms like myelodysplastic syndrome (MDS) is unclear. By data mining with publicly accessible cancer genomics databases and a clinical genomic database from a tertiary medical institution, *DNMT3A* R882 mutations were found to be enriched in AML (53% of all DNMT3A mutations) but decreased in frequency in clonal hematopoiesis of indeterminate potential (CHIP) (10.6%) or other myeloid neoplasms including MDS (27%) (p<.001). Next with the largest cohort of patients with *DNMT3A* R882 mutant MDS known to date from multiple institutions, *DNMT3A* R882 mutant MDS cases were shown to have more severe leukopenia, enriched *SRSF2* and *IDH2* mutations, increased cases with excess blasts (47% vs 22.5%, p=.004), markedly increased risk of AML transformation (25.8%, vs. 1.7%, p=.0001) and a worse progression-free survival (PFS) (median 20.3, vs. >50 months, p=.009) than non-R882 mutant MDS cases. *DNMT3A R882* mutation is an independent risk factor for worse PFS, and importantly the differences in the risk of AML transformation between R882 vs. non-R882 mutant patients cannot be explained by different treatment approaches. Interestingly the higher risk of AML transformation and the worse PFS in *DNMT3A* R882 mutant MDS cases are mitigated by coexisting *SF3B1* or *SRSF2* mutations. The unique clinicopathologic features of *DNMT3A* R882 mutant MDS shed light on the prognostic and therapeutic implications of *DNMT3A* R882 mutations.

## Introduction

As one of the most important tumor suppressors in hematologic malignancies (1–6), DNA methyltransferase enzyme *DNMT3A* is frequently mutated in myeloid neoplasms (MNs) as well as in their early precursors (7–9): its mutations occur in up to 36% of cytogenetically normal acute myeloid leukemia (CN-AML) patients, and 60% of clonal hematopoiesis of indeterminate potential (CHIP) (10, 11). Somatic mutations of *DNMT3A* result in aberrant DNA methylation, disrupting normal hematopoietic stem cell (HSC) differentiation and self-renewal (1, 2, 12), and are associated with adverse overall survival (OS) in AML and myelodysplastic syndrome (MDS) patients (1, 3, 13, 14). Somatic mutations of *DNMT3A* are distributed throughout its coding region with a hot spot at arginine 882 (R882) in MNs. Other pathogenic/likely pathogenic *DNMT3A* mutations include nonsense, frameshift, and missense alterations, presumably resulting in loss-of-functions.

Biochemical and *in vitro* studies suggest that *DNMT3A* R882 mutations have multiple mechanisms of action. Like other loss-of-function mutations, *DNMT3A* R882 mutations display reduced methyltransferase activity on CpG substrates *in vitro* (12, 15, 16), likely due to defective DNA binding and impaired CpG recognition (12) (17, 18), as well as loss of tetramerization (15, 16). In addition, *DNMT3A* R882 mutants associate with wild-type *DNMT3A*, presumably interfering with the whole complex (15, 19) and reduce the overall DNA methyltransferase activity in a dominant-negative manner (17). Moreover, *DNMT3A* R882 mutants may alter substrate preference towards CpG regions with specific flanking sequences, leading to R882 specific hypermethylation in AML patients (20). Other gain-of-function activities include aberrant recruitment of Polycomb Repressive Complexes-1 (PRC1) complex to regulate the expression of genes associated with hematopoietic stem cell differentiation (21), which is a methylation-independent process. These effects of *DNMT3A* R882 mutations are likely not mutually exclusive, resulting in an overall redistribution of DNA methylation in the cancer genome and affect the expression of the downstream target genes.

Although the above-mentioned biochemical and *in vitro* studies suggest *DNMT3A* R882 mutations may have unique biological features in comparison with other non-R882 mutations, it is unclear whether MNs with *DNMT3A* R882 mutations have distinct clinicopathologic features with potential therapeutic implications. The prognostic impact of *DNMT3A* R882 *versus* non-R882 mutations in AML is inconclusive (22–24). With the largest cohort of patients with *DNMT3A* R882 mutated chronic MNs (MDS, MDS/MPN and MPN) known to date, our multi-institution study set out to address whether *DNMT3A* R882 mutations confer unique clinicopathologic features in chronic MNs, specifically MDS. This study could shed light on the mechanistic and clinical implications of different types of *DNMT3A* mutations.

## Methods

### Case Selection

This study was approved by the Institutional Review Boards (IRB) at Mayo Clinic, Rochester Minnesota and six other institutions (Geisinger Medical Laboratories, City of Hope Comprehensive Cancer Center, H. Lee Moffitt Cancer Center, ARUP Laboratories, University of Kansas Medical Center and Yale University) separately. Waivers of informed consent were approved by IRBs based on the retrospective nature of the study and minimal risk to subjects. Patients with *DNMT3A* mutant MDS were identified by querying the clinical genomics database from each institution. Four institutions (H. Lee Moffitt Cancer Center, ARUP Laboratories, University of Kansas Medical Center and Yale University) provided data on *DNMT3A* R882 mutant MDS cases, and 2 (Geisinger Medical Laboratories and City of Hope Comprehensive Cancer Center) along with Mayo Clinic provided all *DNMT3A* mutant MDS cases. A total of 14173 cases sent for myeloid next generation sequencing (NGS) were screened, of which 124 (0.9%) unique MDS cases harbored *DNMT3A* mutations. It should be noted that many cases were sent for myeloid NGS for only diagnostic purposes, and they are not necessarily myeloid neoplasms. Diagnosis was made based on the World Health Organization (WHO) 2017 classification system (25).

### Clinicopathologic Data

Clinical information and follow-up data were obtained from the electronic medical records, including age, gender, complete blood count (CBC), bone marrow morphologic features including blasts percentage and bone marrow cellularity, cytogenetics, co-existing mutations, mutation variant allele frequencies, date of initial diagnosis, status of last follow-up and treatment after initial diagnosis. Cytogenetic status in MDS was classified according to the WHO 2017 classification system (25) into three prognostic groups: very good/good, intermediate, and poor/very poor. M.J., M.A., Y.D., X.Z., P.L., K.Y., M.X., W.C., Y.Z., S.H. and GZ provided acquisition, analysis and interpretation of data, and statistical analysis.

### Next Generation Sequencing Panels

DNA was extracted from fresh bone marrow aspirates and next-generation sequencing (NGS) testing was performed using a targeted NGS next-generation sequencing panel at each institution. The Mayo myeloid NGS panel includes 42 genes commonly mutated in MNs: *ANKRD26*, *ASXL1*, *BCOR*, *CALR*, *CBL*, *CEBPA*, *CSF3R*, *DDX41*, *DNMT3A, ELANE, ETNK1*, *ETV6*, *EZH2*, *FLT3*, *GATA1*, *GATA2*, *IDH1*, *IDH2*, *JAK2*, *KDM6A*, *KIT, KRAS*, *MPL*, *NPM1*, *NRAS*, *PHF6*, *PTPN11*, *RAD21*, *RUNX1*, *SETBP1*, *SH2B3*, *SF3B1*, *SRP72*, *SMC3*, *SRSF2*, *STAG2*, *TERT*, *TET2*, *TP53*, *U2AF1, WT1*, and *ZRSR2*. The library preparation, sequencing and data analysis were described before (26). Briefly, libraries were prepared using the Agilent SureSelect‐XT Target Enrichment Kit (SureSelectXT, Agilent, Santa Clara, CA). and sequencing was performed on MiSeq or HiSeq platforms (Illumina, San Diego, CA) at the Mayo Clinic Clinical Genome Sequencing Laboratory. Different panels from other institutions were described in **Supplementary Table 1**, which are all clinical NGS assays performed in CLIA-CAP approved laboratories.

### Genomics Data Analysis

Targeted next generation sequencing data from 75191 unique samples in the GENIE database (v.8.0, access date: April 10, 2020), and 6764 samples from 6 nonduplicate myeloid studies available in cBioPortal (access date: April 10, 2020) were retrieved. Both GENIE and cBioPortal databases are publicly accessible (27, 28). We removed duplicated patients and only kept their earliest record and identified a total number of 1122 AML and 312 MDS cases with *DNMT3A* mutations. To analyze the pattern of coexisting genes of interest (see **Supplementary Table 2** for gene lists), we used an approach similar to what had been described previously (29). Distributions of samples based on *DNMT3A* alteration status (R882 and non-R882 mutations) and alteration status of genes of interest were summarized in 2 × 2 contingency tables. Single nucleotide variants (SNVs) and insertions/deletions (indels) were included in the analysis. Odds ratios (ORs) were calculated for each co-mutated gene. The χ2 test was used with the 2 × 2 contingency tables to test whether alterations coexist or are mutually exclusive to each other. The 95% CIs of ORs were calculated as exp(ln(OR) ± 1.96*SE(ln(OR))), where SE(ln(OR)) =√1/A + 1/B + 1/C + 1/D (https://www.statology.org/odds-ratio-relative-risk-excel/). Benjamini-Hochberg correction method was used to adjust for multiple testing.

### Statistical Analysis

Statistical significance in different groups was determined using Fisher’s exact test with a contingency table. Survival analysis, Cox proportional hazards model analysis, Fisher’s exact test, ROC analysis and t-tests were done using BlueSky statistics package (https://www.blueskystatistics.com/Default.asp). A p value less than 0.05 was considered statistically significant. Progression-free survival (PFS) is defined as the time from MDS diagnosis to either AML transformation or death.

### Data Sharing Statement

The datasets used and/or analyzed during the current study are available from the corresponding author.

## Results

**Figure 1:**
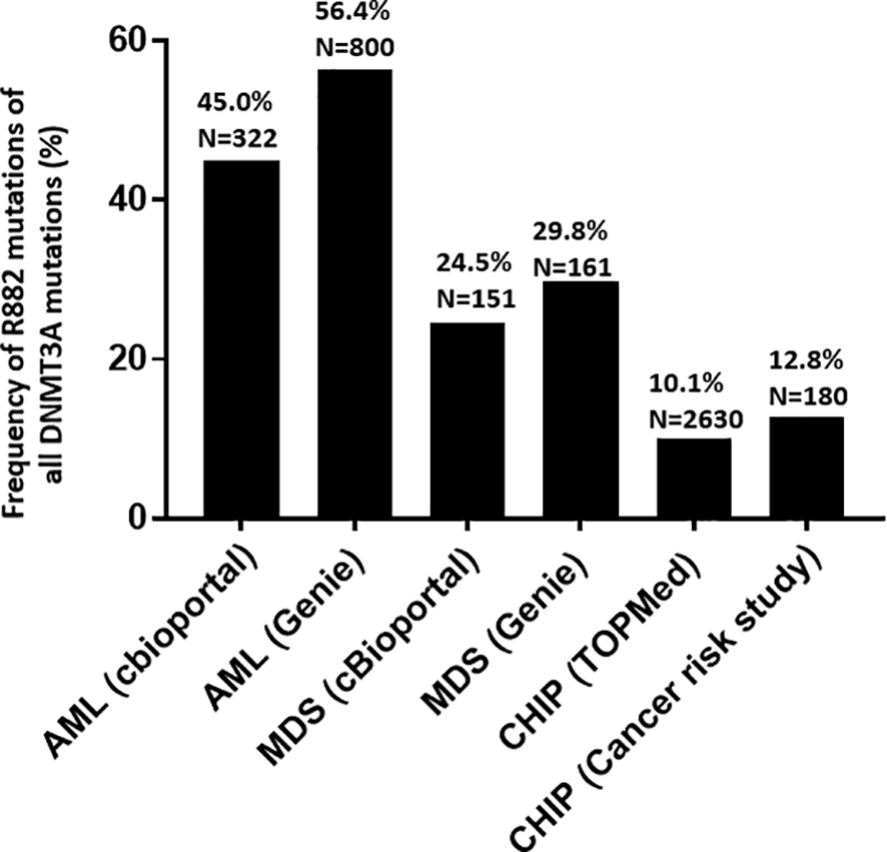
Frequency and distribution of *DNMT3A* mutations (R882 *versus* non-R882) in acute myeloid leukemia (AML), myelodysplastic syndrome (MDS), and clonal hematopoiesis of indeterminate potential (CHIP). GENIE database and myeloid studies available in cBioPortal were used for to retrieve DNMT3A mutations in patients with myeloid neoplasms. Two studies (7, 30) on clonal hematopoiesis of indeterminate potential (CHIP) were used to retrieve DNMT3A mutations in healthy individuals without hematologic malignancies.

Using publicly accessible genomics databases (27, 28), the fractions of R882 mutations among all *DNMT3A* mutations in various myeloid disease states were compared. The GENIE database and myeloid studies available in cBioPortal were used to retrieve *DNMT3A* mutations in patients with MNs. Two large studies (7, 30) on CHIP were used to retrieve *DNMT3A* mutations in individuals without hematologic malignancies. *DNMT3A* R882 mutations showed different distributions in different myeloid disease states (**Figure 1**). The overall fraction of R882 mutations among all *DNMT3A* mutations is much higher in AML (53%) than in CHIP (10.6%) (*p*<.0001 by Fisher’s exact test). Given that *DNMT3A* mutations often occurred early in the development of myeloid neoplasm and rarely acquired during disease progression (31–34), this result suggests that CHIP harboring R882 mutations has a higher risk of developing AML than non-R882 mutations (about 5-fold). Interestingly, the fraction of *DNMT3A* R882 mutations of all *DNMT3A* variants in MDS fell between that of CHIP and AML (27%, *p*<.001 when compared with either CHIP or AML). Given that MDS is a myeloid neoplasm with the potential of AML transformation, it suggests that R882 mutations could also confer a higher risk of AML transformation than other *DNMT3A* mutations in MDS. Similarly, patients with CHIP harboring *DNMT3A* R882 mutation may have a higher risk of developing MDS than CHIP with other *DNMT3A* mutations.

**Figure 2:**
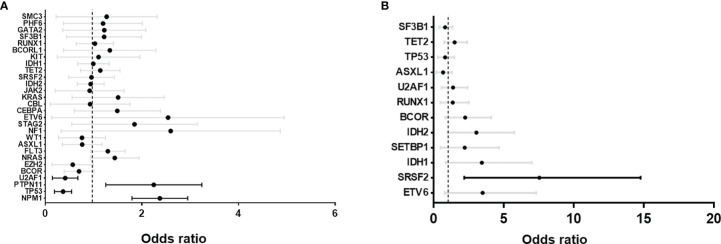
Forrest plots of odds ratio with 95% confidence internal of co-existing mutations between *DNMT3A* R882 vs. non-R882 mutant cases. **(A)** Acute myeloid leukemia (AML) and **(B)** myelodysplastic syndrome (MDS). GENIE database and myeloid studies available in cBioPortal were extracted and used for the analysis. Statistically significant co-existing mutation are highlighted with black lines.

We then selected a list of commonly mutated genes in AML and MDS (**Supplementary Table 2**) and compared the coexisting mutations between *DNMT3A* R882 mutant and non-R882 mutant AML and MDS cases respectively using data from GENIE and cBioPortal. Only genes with at least 5 co-mutations were included (**Figure 2**). Mutations of *NPM1* (odds ratio 2.3, range: 1.8-2.3) and *PTPN11* (odds ratio 2.1, range: 1.3-3.3) were enriched in *DNMT3A* R882 mutant AMLs (**Figure 2A**), while *TP53* (odds ratio 0.3, range: 0.2-0.6) and *U2AF1* (odds ratio 0.4, range: 0.2-0.7) were more frequently co-mutated in non-R882 mutant AML cases (**Figure 2B**). In MDS, mutations of *SRSF2* (odds ratio 5.7, range: 2.2-14.8) and *IDH2* mutations were more enriched with *DNMT3A* R882 mutations (odds ratio 2.4, range: 1.0-5.8) (**Figure 2B**). These distinct co- mutation patterns suggest possible unique biologic features of *DNMT3A* R882 bearing MNs.

The different distributions of *DNMT3A* R882 mutations between AML and MDS were also confirmed with clinic data from Mayo clinic. Of 2926 cases with a myeloid NGS test performed, 79 patients with *DNMT3A* mutant MNs were identified, including 40 (51%) AML, 28 (35%) MDS, 3 (4%) MDS/MPN (myelodysplastic syndrome/myeloproliferative neoplasm), and 8 (10%) MPN. Our data is concordant with the results from cancer genomics database analysis which show that, among *DNMT3A* mutated MNs, the *DNMT3A* R882 mutations are mostly enriched in AML (67.5%, *p<*.001 by Fisher’s exact test); compared with 25% in MDS (*p*=.009 by Fisher’s exact test), and 12.5% in MPN (*p*=.06 by Fisher’s exact test) (**Table 1**).

**Table 1:** Demographics and disease presentation of myeloid neoplasms with *DNMT3A* (R882 vs. Non-R882) mutations.

	*DNMT3A* R882 (Total = 36)	*DNMT3A* non-R882 (Total = 43)	*P* value*
**Average age (Range)**	66 (40–91)	72 (47-86)	NS
**Gender**			
Male	21	17	NS
Female	15	26	
**Disease at presentation**			
AML (total 40)	27 (67.5%)	13 (32.5%)	0.0003
MDS (total 28)	7 (25%)	21 (75%)	0.009
MDS/MPN (total 3)	1 (33.5%)	2 (66.5%)	NS
MPN (total 8)	1 (12.5%)	7 (87.5%)	0.06

*T-test for age, and Fisher’s exact test for others.

NS, not significant; AML, Acute myeloid leukemia; MDS, Myelodysplastic syndrome; MPN, Myeloproliferative neoplasm.

**Figure 3:**
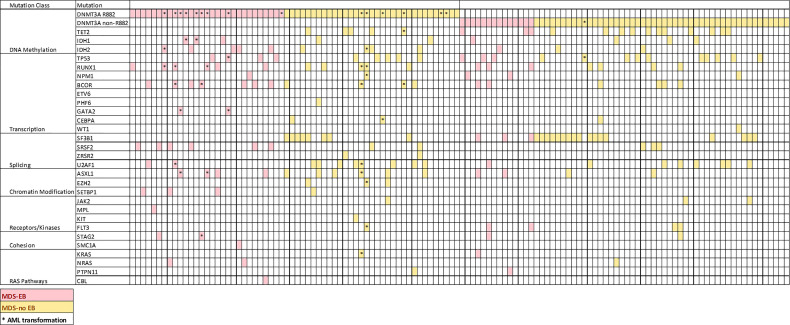
Distribution of coexisting mutations of genes commonly mutated in AML and MDS ordered by gene function. Each column represents a patient.

We then assessed the clinicopathologic features of the chronic MNs harboring specific *DNMT3A* R882 mutations. Only 9 patients with a chronic myeloid neoplasm harboring *DNMT3A* R882 mutations were identified in the Mayo cohort, so we expanded our study to 6 additional institutions as described in Methods. From a total of 14173 patients with myeloid NGS performed from seven institutions, 170 patients with *DNMT3A* mutant chronic MNs (MDS, MDS/MPN and MPN) were identified, including 124 patients with MDS. AML patients were not included given the studies published (22–24). Given most of the chronic MNs harboring *DNMT3A* R882 mutations identified were MDS patients, we focused further analysis on this group. As expected, non-R882 mutations are scattered in distribution and there are rare sites mutated more than 2 times (**Supplemental Figure 1**). A comparison of the clinicopathologic features between *DNMT3A* R882 vs. non-R882 groups was shown in **Table 2**. No significant differences were observed in age, gender, mutation variant allele frequency (VAF), or bone marrow cellularity between the two groups. CBC data was available for 91 patients. No significant differences in anemia (76% vs. 67.5%, p>0.05) or thrombocytopenia (59% vs. 51%, p>0.05) were observed between patients with R882 mutant and non-R882 mutant MDS cases. Interestingly more patients in the *DNMT3A* R882 mutant groups showed leukopenia (WBC<2 x 10^9/^L) than the non-R882 groups (31% vs. 24%), and the *DNMT3A* R882 mutant MDS cases show lower average WBC level than non-R882 mutant cases (mean: 3.0×10^9^/L vs. 4.4×10^9^/L, *p*=.02) (**Table 2**). Consistent with the cancer genomics data analysis (**Figure 2B**), *SRSF2* mutations were enriched in the *DNMT3A* R882 mutant group compared to the non-R882 mutant group (14.5% vs. 4.8%, *p*=.03), and mutations of *IDH2* and *BCOR* also show a trend of enrichment in the *DNMT3A* R882 mutant group (14.5% vs. 4.8%, *p*=.05 for *IDH2*, 17.7% vs. 9.6%, *p*=.06 for *BCOR*) in comparison to the non-R882 group (**Figure 3**).

**Table 2:** Summary of the clinicopathologic features of *DNMT3A* (R882 vs. Non-R882) mutations in myelodysplastic syndromes.

	*DNMT3A* R882 (62)	*DNMT3A* Non-R882 (62)	P Value
**Average age (range)**	67 (29-91)	69 (23-86)	NS
**Gender**	39 M/23 F	32 M/30 F	NS
CBC*			
Anemia (Hg <10 g/dL)	41/54 (76%)	25/37 (67.5%)	NS
Leukopenia (WBC<2 x 10^9/^L)	17/54 (31%)	9/37 (24%)	NS***
Thrombocytopenia (Plt <100 x 10^9/^L)	32/54 (59%)	19/37 (51%)	NS
**MDS-EB**	29 (47%)	14 (22.5%)	0.004
**Average bone marrow blast % (range)**	8 (0-20)	1 (0-20)	
Cytogenetic Risk Group**			
Very good- good	30/54 (55.5%)	39/58 (67%)	NS
Intermediate	11/54 (20%)	6/58 (10%)	NS NS
Poor-very poor	13/54 (24%)	13/58 (22%)	
**Average VAF%^#^**	27 (2-49)	28 (5-66)	NS
**Ring Sideroblasts**	9/62 (35%)	15/62 (24%)	NS
**Progressed to AML**	15/58 (25.8%)	1/60 (1.7%)	0.0001
**Survival^$^**			
Dead	29/58 (50%)	18/60 (30%)	0.02
Alive	29/58 (50%)	42/60 (70%)	

M, male; F, Female; CBC, Complete blood counts; Hg, hemoglobin; WBC, white blood count; Plt, platelets; MDS-EB, Myelodysplastic syndrome with excess blasts; VAF, Variant allele frequency; AML, Acute myeloid leukemia; NS, not significant.

*CBC not available for 33 patients (8 R882, 25 non-R882),

**Cytogenetic data not available for 12 patients.

^#^VAF not available for 14 patients.

^$^Survival data not available for 6 patients.

***p = .02 by t-test comparing average white blood cell counts.

**Figure 4:**
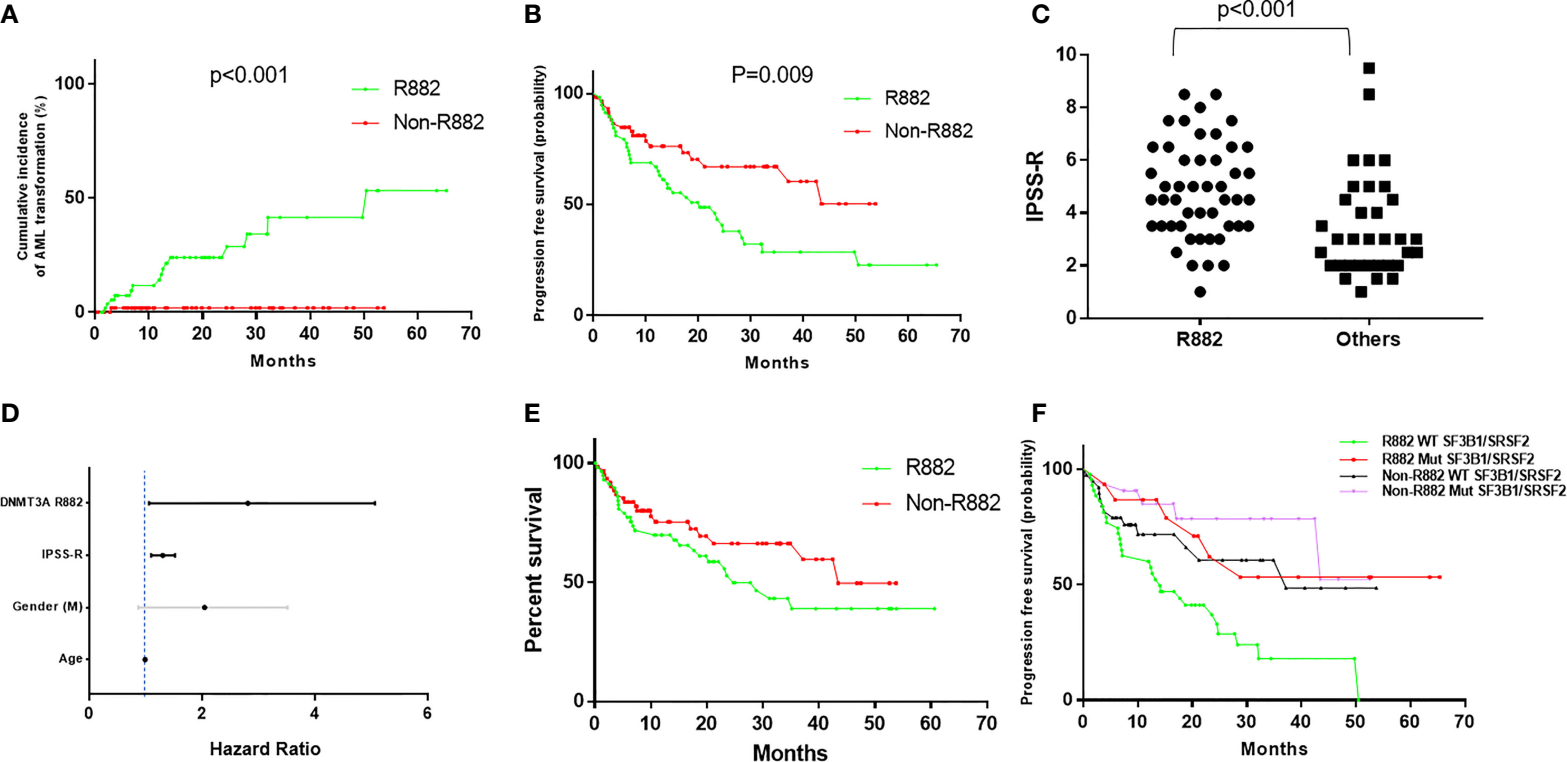
*DNMT3A* R882 mutations confer increased risk of AML transformation and worse progression free survival in MDS, which are masked by the coexisting SF3B1 or SRSF2 mutations. **(A)** Cumulative incidence of AML transformation in *DNMT3A* (R882 vs. Non-R882) mutant MDS. **(B)** Progression free survival analysis in patients with *DNMT3A* (R882 vs. Non-R882) mutant myelodysplastic syndrome (MDS). **(C)** The distribution of IPSS-R scores in the R882 group and the other group. **(D)** Cox proportional hazards model analysis of risk factors for worse progression free survival. Statistically significant factors are highlighted with black lines. **(E)** Overall survival analysis in patients with *DNMT3A* (R882 vs. Non-R882) mutant myelodysplastic syndrome (MDS). **(F)** Progression free survival analysis in *DNMT3A* (R882 *vs.* Non-R882) mutant MDS with mutant and wildtype *SF3B1/SRSF2*.

**Figure 5:**
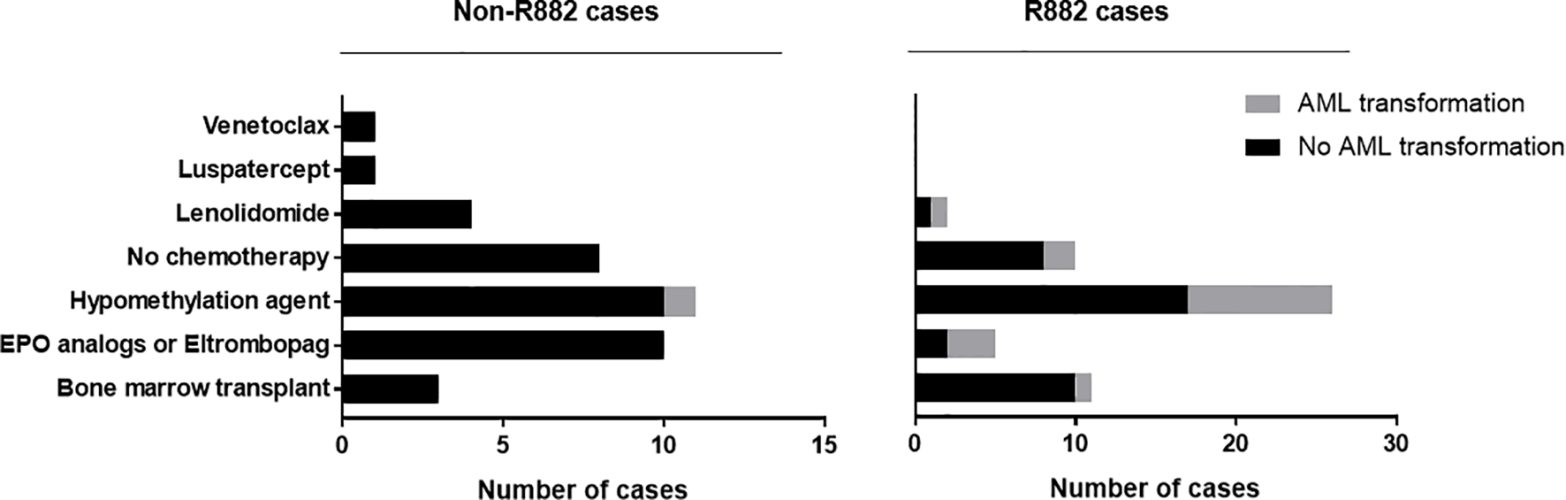
MDS treatment approaches and AML transformation. Treatment information was collected for 53 out of 62 MDS patients with DNMT3A R882 mutations, and 38 out of 62 patients with DNMT3A non-R882 mutations. Different treatment approaches were listed together with the occurrence AML transformation.

Importantly, patients with R882 mutant MDS were more likely to present with excess blasts (47% vs 22.5%, *p*=.004), which could not be explained by the differences of cytogenetic risks (**Table 2**): of 112 patients with available cytogenetic data, no difference in cytogenetic risk between different groups was observed (55.5% vs. 67%, p>0.05 for very good or good; 20% vs. 10%, p>0.05 for intermediate and 24% vs. 22%, p>0.05 for poor to very poor cytogenetic category for R882 and non-R882 groups respectively). Follow up data was available for 116 patients with 8 lost to follow-up (range 0 – 65.4 months, median 14.5). MDS cases harboring *DNMT3A* R882 mutations showed significantly higher risk of AML transformation (25.8%, vs. 1.7%, *p*=.0001) than the non-882 group (**Figure 4A**), which is not entirely due to increased frequency of excess blasts, as MDS-EB cases harboring *DNMT3A* R882 mutations also showed significantly higher risk of AML transformation than the non-R882 group (34.5%, vs. 0%, *p*=.01). Interestingly the same is also true for MDS without EB (18.2% vs. 2%, *p*=.01), supporting that *DNMT3A* R882 confer an increased risk of AML transformation independent of blast count. Survival analysis also supported that *DNMT3A* R882 mutations confer a higher risk in MDS. Patients with a *DNMT3A* R882 mutation showed a worse progression free survival (PFS) (median 20.3, vs. >50 months, *p*=.009) than those with non-R882 mutations (**Figure 4B**). Interestingly there is no PFS difference between MDS cases with DNMT3A truncating mutations and other missense mutations (**Supplementary Figure 3**). During the follow-up, 29 (50%) of those patients with R882 mutations were dead, in comparison to 18 (30%) with non-R882 mutations (*p*=.04 by Fisher’s exact test). IPSS-R scores were available for 82 patients, and *DNMT3A* R882 mutations are associated with higher IPSS-R scores (p<0.001 by ROC analysis) (**Figure 4C**), which is mostly due to increased blasts (**Table 2**). Importantly, multivariate Cox proportional hazards model analysis shows *DNMT3A* R882 mutation is an independent risk factor for worse PFS (hazard ratio: 2.3, confidence interval: 1.1-5.1) in addition to the IPSS-R scores (hazard ratio: 1.3, confidence interval: 1.1-1.5) (**Figure 4D**). The VAF distribution of DNMT3A mutations ranges from 1.7% to 66% (**Supplementary Figure 2**) with most cases with a VAF of more than 10%. Importantly VAFs of DNMT3A mutations have no correlation with PFS (p=0.6 by Cox proportional hazards model analysis). Consistently, the overall survival in MDS was shorter in those who harbored R882 mutations in contrast to those with non-R882 mutations (median survival 24.7 vs. 43.4 days), though did not reach statistical significance (*p*=.1) most likely due to the size of the cohort (**Figure 4E**). We took a close look at the factors associated with AML transformation. Patients who underwent AML transformation tended to be older (mean 74 vs 66, *p*=.01, t-test) in the *DNMT3A* R882 mutant group. We further studied cases with coexisting mutations involving SF3B1 and SRSF2 as they are common splicing factor genes mutated in MDS. Interestingly, none of the *DNMT3A* R882 mutant cases with coexisting *SF3B1* or *SRSF2* mutations had AML transformation (33% vs. 0%, *p*=.03 by Fisher’s exact test when combining both). In addition, the *DNMT3A* R882 mutant cases without coexisting *SF3B1* or *SRSF2* mutations have much worse PFS than those with coexisting *SF3B1* or *SRSF2* mutations (Median 14.1 months vs. >50, *p*=.0009), which has similar PFS as the *DNMT3A* non-R882 mutant cases (**Figure 4F**). To assess whether different treatment approaches explain different risk of AML transformation, treatment information was summarized together with the occurrence of AML transformation (**Figure 5**). Treatment histories were available for 53 out of 62 patients with *DNMT3A* R882 mutations, and 38 out of 62 patients with *DNMT3A* non-R882 mutations. AML transformation occurs in patients with different treatment strategies, and the differences in the risk of AML transformation between R882 vs. non-R882 mutant patients cannot be explained by different treatment approaches (**Figure 5**).

## Discussion

Precision oncology requires variant level understanding of the somatic mutations: different mutations of the same gene may show different mechanisms of action, confer different clinicopathologic features, and have different therapeutic and clinical implications. Biochemically compared with other *DNMT3A* mutations, R882 mutations show additional dominant-negative and gain-of-function activities, suggesting different biological mechanism of action and possible unique clinicopathologic features in *DNMT3A* mutant MNs. Given *DNMT3A* R882 mutations are acquired early in the precursors of MNs, significantly higher fractions of *DNMT3A* R882 mutations in AML than MDS in our study suggests that these mutations may confer higher risk of developing AML than non-R882 mutations. Indeed, MDS cases harboring *DNMT3A* R882 mutations tend to have increased blasts and a higher risk of AML transformation compared with non-R882 group. Our results demonstrate that mutation type contributes to the heterogeneity of clinical outcomes of *DNMT3A* mutant MNs (3, 15, 35), and the *DNMT3A* R882 mutations have a stronger leukemogenic potential than other *DNMT3A* mutations, possibly due to greater reductions in methyltransferase activity than non-R882 mutations (15, 16) and other gain-of-function activities. It should be noted that the prognostic significance of DNMT3A R882 vs. non-R883 mutations depends on the disease context, as the prognostic impact of *DNMT3A* R882 *versus* non-R882 mutations in AML is inconclusive (22–24).

With population-based cohorts it was shown that CHIP confers an increased relative risk of myeloid malignancies at a rate of about 0.5 to 1% per year (7, 8). There are different factors influencing the risk of progression, including the specific gene(s) that are mutated (36). *DNMT3A* is the most mutated gene in CHIP, and its mutations overall predict only a moderately elevated risk of leukemic progression (12, 37, 38). Our study shows that not all *DNMT3A* mutations are created equal, for example, significantly higher frequency of R882 mutation of all DNMT3A mutations in AML than CHIP (**Figure 1**) suggests that R882 mutations may confer a higher risk of developing AML than other non-R882 mutations in CHIP. Thus, CHIP with *DNMT3A* R882 should probably be considered as higher-risk and be followed up more frequently than CHIP with non-R882 mutations. In addition, CHIP has been shown to be associated with cardiovascular disease including atherosclerosis, thus additional studies are needed to assess *DNMT3A* R882 mutations’ risk for cardiovascular diseases and atherosclerosis, and whether reduction of modifiable cardiovascular risk factors is needed in this group. Increased understanding of the molecular and clinical implications of *DNMT3A* mutations in CHIP may lead to better patient stratification algorithms (12, 37, 38).

MDSs are considered precursor diseases for AML; 25% of patients with MDS will develop AML during their disease course (39). MDS patients with *DNMT3A* mutations had a higher risk of leukemia transformation and shorter overall survival (6, 31). Our study suggests that different *DNMT3A* mutations have different clinical implications, and *DNMT3A* R882 mutations are important prognostic markers in this setting. The higher risk of AML transformation compared to non-R882 mutant cases suggests that *DNMT3A* R882 mutant MDS cases, even ones without excess blasts at initial diagnosis, probably should be managed differently from non-R882 mutant MDS cases. However, coexisting mutations should also be considered in risk stratification of patients. Secondary mutations may dictate the phenotype of the neoplasm. For example, *DNMT3A* mutation is often followed by a mutation in *NPM1*, *FLT3*, or *IDH1* during AML development (19). Our study shows that in the presence of *SF3B1* and *SRSF2* mutations, none of the *DNMT3A* mutant MDS cases had AML transformation, suggesting these mutations are disease and phenotype-defining in the context of *DNMT3A* R882 mutations. Consistent with this notion, it has been shown that patients with wild type *SF3B1* and mutant *DNMT3A* mutations had an inferior overall survival and higher risk of AML transformation than ones with mutant *SF3B1* and mutant *DNMT3A* (40), and *DNMT3A* mutations predicted worse prognosis in the *SF3B1*-wild type patients but not *SF3B1*-mutated patients (31).

The mechanisms of action of and the unique clinicopathologic features conferred by *DNMT3A* R882 mutations also have therapeutic implications. Patients heterozygous for the DNMT3A R882 mutation also have wild type *DNMT3A*, the function of which may be improved if the R882 form was selectively inhibited. Indeed, mechanistically it was shown that mutation of the heterodimerization motif that interferes with R882 mutant *DNMT3A* binding to wild type *DNMT3A* proteins partially reversed the CpG hypomethylation caused by R882 mutant *DNMT3A* (41). In addition, DNA methyltransferase inhibitor 5-azacytidine and decitabine are commonly used for the treatment of MDS. Although it was shown that patients with a *DNMT3A* mutation were more likely to have a favorable response to hypomethylating therapy used in the treatment of MDS, MDS/MPN and secondary AML (42), the conclusion was not supported by other studies (43, 44). It is also unclear how patients with different *DNMT3A* mutations (R882 mutations vs. others) respond to hypomethylating agents. Given *DNMT3A* R882 mutations confer markedly decreased methyltransferase activity and dominant-negative function, it is important to separate R882 from non-R882 mutant groups in assessing their responses to hypomethylation agents.

Our study however has limitations, and questions remain to be addressed. First, our cohort was from 7 different medical institutions. Given the different sources of data, only some of the patients’ treatment history was available. It would be important to assess the complete treatment histories and treatment responses of DNMT3A R882 vs. non-R882 mutant MDS to different therapies, including hypomethylation agents. Secondly, a longitudinal study is needed to confirm the risk of developing AML in patients with CHIP harboring *DNMT3A* R882 mutations. And finally, the number of other chronic MNs other than MDS is too low to assess their clinicopathologic features; hence a larger cohort is needed for complete characterization.

Overall, our study shows that *DNMT3A* R882 mutations confer unique clinicopathologic features with an increased risk of AML transformation in MDS, which is modified by the coexisting SF3B1 or SRSF2 mutations. The study sheds light on the prognostic and therapeutic implications of different types of *DNMT3A* mutations.

## Data Availability Statement

The raw data supporting the conclusions of this article will be made available by the authors, without undue reservation.

## Ethics Statement

The studies involving human participants were reviewed and approved by The Institutional Review Boards (IRB) at Mayo Clinic. Written informed consent for participation was not required for this study in accordance with the national legislation and the institutional requirements.

## Author Contributions

MJ, GZ, MA, YD, RH, and XZ performed study concept and design. GZ, AA-K, DV, DC and MJ performed development of methodology and writing, review, and revision of the paper. MJ, MA, YD, XZ, PL, KY, MX, WC, YZ, SH, and GZ provided acquisition, analysis and interpretation of data, and statistical analysis. All authors contributed to the article and approved the submitted version.

## Conflict of Interest

The authors declare that the research was conducted in the absence of any commercial or financial relationships that could be construed as a potential conflict of interest.

## Publisher’s Note

All claims expressed in this article are solely those of the authors and do not necessarily represent those of their affiliated organizations, or those of the publisher, the editors and the reviewers. Any product that may be evaluated in this article, or claim that may be made by its manufacturer, is not guaranteed or endorsed by the publisher.
