# Supplementary figures and images for: DNMT3A R882 Mutations Confer Unique Clinicopathologic Features in MDS Including a High Risk of AML Transformation
Source: Front Oncol. 2022 Feb 28;12:849376. doi: 10.3389/fonc.2022.849376 (PMC8918526; doi:10.3389/fonc.2022.849376)

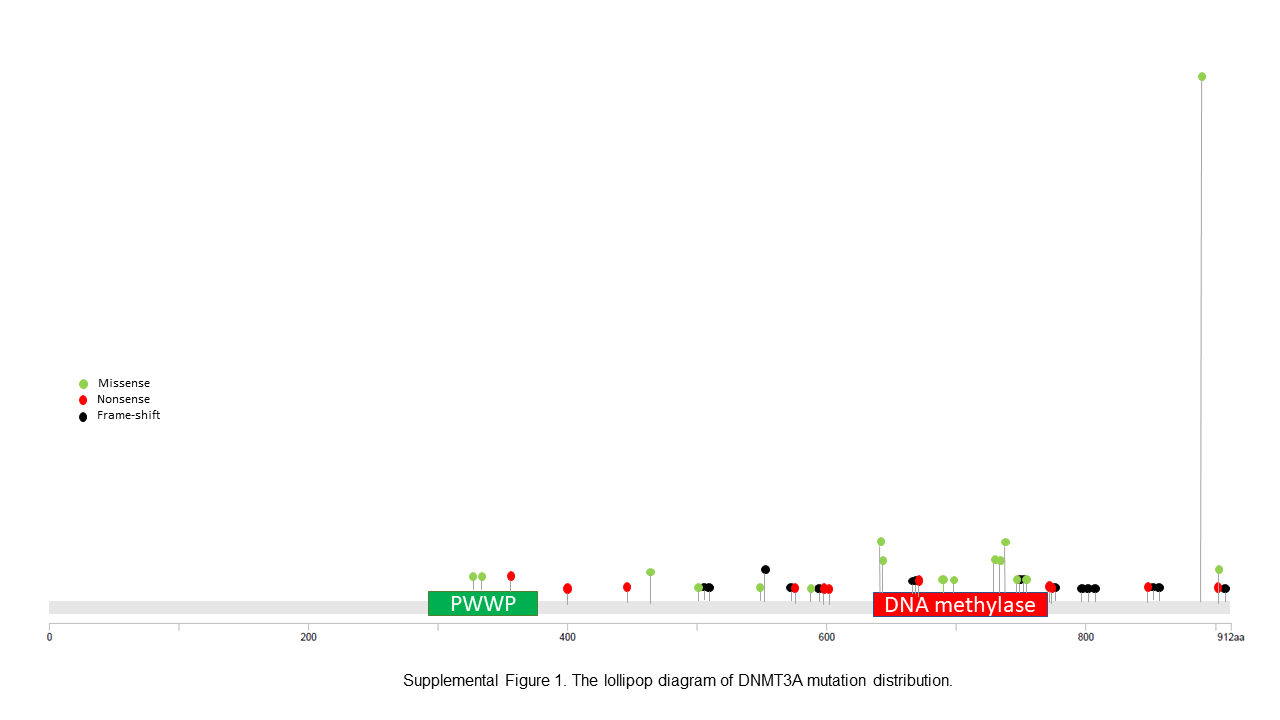

Supplement: Supplementary file 1 [file Image_1.tif]

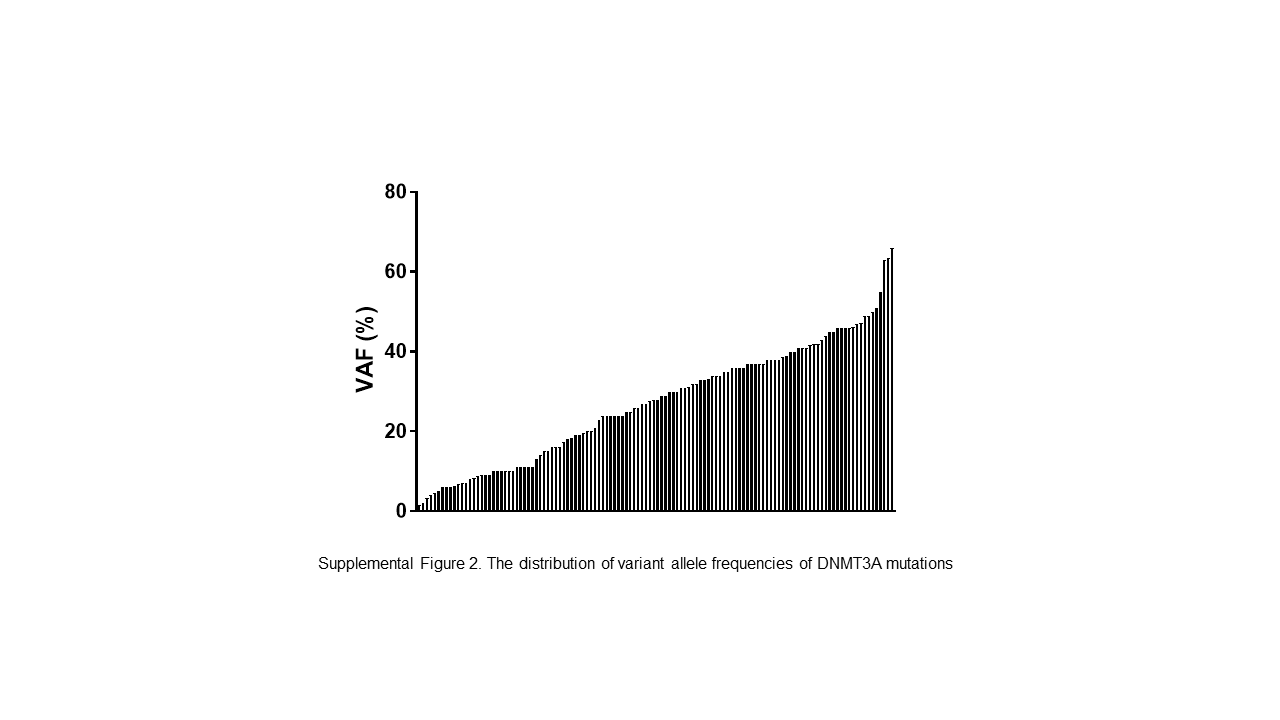

Supplement: Supplementary file 2 [file Image_2.tif]

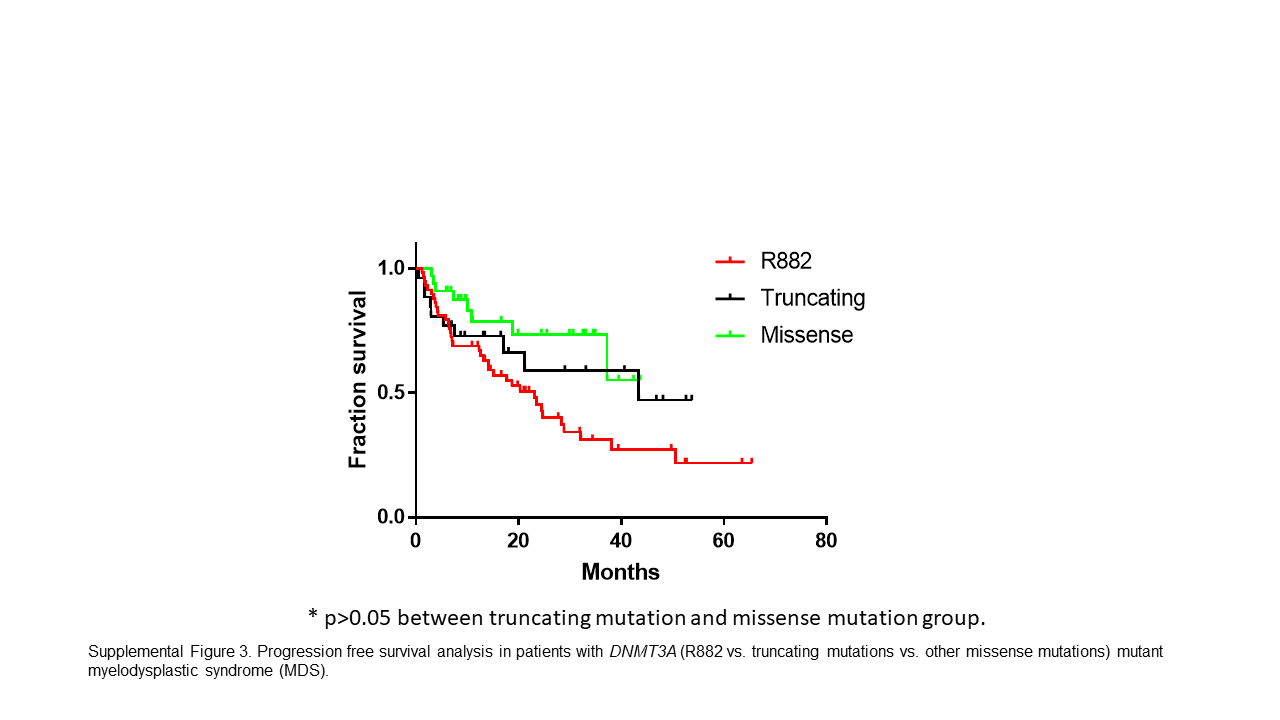

Supplement: Supplementary file 3 [file Image_3.tif]
