# Supplementary material for: DNMT3A R882 Mutations Confer Unique Clinicopathologic Features in MDS Including a High Risk of AML Transformation
Source: Front Oncol. 2022 Feb 28;12:849376. doi: 10.3389/fonc.2022.849376 (PMC8918526; doi:10.3389/fonc.2022.849376)
Supplement: Supplementary file 4 [file Table_1.docx]

| **Institutions** | **NGS panel** |
| --- | --- |
| Geisinger | ABL1, ANKRD26, ASXL1, ATRX, BCOR, BCORL1, BRAF, BTK, CALR, CBL, CBLB, CBLC, CCND2, CDC25C, CDKN2A, CEBPA, CSF3R, CUX1, CXCR4, DCK, DDX41, DHX15, DNMT3A, ENTK1, ETV6, EZH2, FBXW7, FLT3, GATA1, GATA2, GNAS, HRAS, IDH1, IDH2, IKZF1, JAK2, JAK3, KDM6A, KIT, KMT2A, KRAS, LUC7L2, MAP2K1, MPL, MYC, MYD88, NF1, NOTCH1, NPM1, NRAS, PDGFRA, PHF6, PPM1D, PTEN, PTPN11, RAD21, RBBP6, RPS14, RUNX1, SETBP1, SF3B1, SH2B3, SLC29A1, SMC1A, SMC3, SRSF2, STAG2, STAT3, TET2, TP53, U2AF1, U2AF2, WT1, XPO1, and ZRSR2. |
| Moffitt | ABL1, ASXL1, ATRX, BCOR, BCORL1, BRAF, CALR, CBL, CBLB, CBLC, CDKN2A, CEBPA, CSF3R, CUX1, DNMT3A, ETV6, EZH2, FBXW7, FLT3, GATA1, GATA2, GNAS, HRAS, IDH1, IDH2, IKZF1, JAK2, JAK3, KDM6A, KIT, KMT2A, KRAS, MPL, MYD88, NOTCH1, NPM1, NRAS, PDGFRA, PHF6, PTEN, PTPN11, RAD21,  RUNX1, SETBP1, SF3B1, SMC1A, SMC3, SRSF2, STAG2, TET2, TP53, U2AF1, WT1 and ZRSR2 |
| City of hope | ABL1, ARID1A, ASXL1, ATM, B2M, BCL2, BCL6, BCOR, BCORL1, BIRC3, BRAF, BTK, CALR, CARD11, CBL, CBLB, CCND1, CCND3, CD38, CD3E, CD3G, CD79A, CD79B, CDK4, CDK7, CDKN1B, CDKN2A, CDKN2B, CDKN2C, CEBPA, CHD2, CRBN, CREBBP, CSF3R, CUX1, CXCR4, DDx3x, DIS3, DNMT3A, E2F1, EGFR, EP300, ETV6, EZH2, FBXW7, FGFR3 , FH, FLT3, FAM46C, FOXO1, GATA1, GATA2, GNA13, GNAS, HCK, HRAS, ID3, IDH1, IDH2, IGLL5, IKZF1, IL6, IL7R IRF4, JAK1, JAK2, JAK3, KDM6A, KIT, KMT2A, KMT2C, KMT2D, KRAS, LCK, LMO2, MAPK1, MEF2B, MGA, MIR142, MPL, MYC, MYD88, NFKB2, NOTCH1, NOTCH2, NPM1, NRAS, NTRK1, PAX5, PDCD1, PDGFRA, PHF6, PIGA, PIK3CA, PIK3CD, PIK3CG, PIK3R5, PLCG2, POT1, PRDM1, PTEN, PTK2B, PTPN11, RAD21, RB1, RFC4, RPS15, RUNX1, SETBP1, SF3B1, SMC1A, SMC3, SOCS1, SOCS6, SPI1, SRSF2, STAG2, STAT3, STAT5A, STAT5B, STAT6, SUZ12, TCF3, TET2, TNFAIP3, TP53, TP63, U2AF1, UBR5, WHSC1, WHSC1L1, WT1, XPO1, ZEB1, ZRSR2 |
| Yale | ABL1, ALK, ASXL1, ATRX, BCOR, BCORL1, BRAF [partial sequencing of gene regions with known mutations], BRCC3, CALR [partial sequencing of gene regions with known mutations], CBL, CEBPA, CSF3R, DNMT3A, EED, EP300, ETV6, EZH2, FLT3 [partial sequencing of gene regions with known mutations], GATA1, GATA2, IDH1 [partial sequencing of gene regions with known mutations], IDH2 [partial sequencing of gene regions with known mutations], JAK2, KIT, KRAS, MPL, MYC, NF1, NPM1 [partial sequencing of gene regions with known mutations], NRAS, PDGFRA, PDS5B, PHF6 PRPF8, PTPN11, RAD21, RUNX1, SETBP1, SF3B1, SMC1A, SMC3, SRSF2, STAG1, STAG2, TET2, TP53, U2AF1, WT1, ZRSR2 |
| ARUP | ANKRD26, ASXL1, ASXL2, BCOR, BCORL1, BRAF, CALR, CBL, CBLB, CEBPA, CSF3R, CUX1, DDX41, DNMT1, DNMT3A, ELANE, ETNK1, ETV6, EZH2, FBXW7, FLT3, GATA1, GATA2, GNAS, HNRNPK, IDH1, IDH2, IL7R, JAK1, JAK2, JAK3, KDM6A, KIT, KMT2A, KRAS, LUC7L2, MPL, NOTCH1, NPM1, NRAS, NSD1, PHF6, PIGA, PRPF40B, PRPF8, PTPN11, RAD21, RUNX1, SETBP1, SF3B1, SH2B3, SMC1A, SMC3, SRSF2, STAG2, STAT3, STAT5B, SUZ12, TET2, TP53, U2AF1, U2AF2, WT1, ZRSR2 |
| KUMC | ABL1, ADA, ANKRD26, ASXL1, ASXL2, ATM, ATRX, BCL6  BCOR, BCORL1, BCR, BIRC3, BLM, BRAF, BRCA1, BRCA2, BRINP3, C17orf97, CALR, CARD11, CBL, CBLB, CBLC, CDKN2A  CEBPA, CHEK2, CREBBP, CRLF2, CSF1R, CSF3R, CTCF, CUX1, DAXX, DDX41, DNM2, DNMT1, DNMT3A, EED, EGFR, ELANE  EP300, ETNK1, ETV6, EZH2, FAM154B, FAM47A, FAS, FBXW7  FLRT2, FLT3, GATA1, GATA2, GJB3, GNAS, HNRNPK, HRAS  IDH1, IDH2, IKZF1, IKZF3, IL7R, JAK1, JAK2, JAK3, KAT6A  KCNA4, KCNK13, KDM6A, KDR, KIT, KLHDC8B, KLHL6, KMT2A, KMT2C, KRAS, LRRC4, LUC7L2, MAP2K1, MLH1, MPL  MSH2, MSH6, MYC, MYD88, NBN, NF1, NOTCH1, NPAT, NPM1  NRAS, NSD1, NTRK3, OR13H1, OR8B12, P2RY2, PAX5, PCDHB1  PDGFRA, PHF6, PML, PMS2, PRAMEF2, PRF1, PRPF40B, PRPF8, PTEN, PTPN11, RAD21, RB1, RELN, RUNX1, SETBP1, SF1, SF3A1, SF3B1, SH2B3, SH2D1A, SMARCB1, SMC1A, SMC3  SRP72, SRSF2, STAG2, STAT3, STXBP2, SUZ12, TAL1, TERC  TERT, TET2, TNFRSF13B, TP53, TPMT, TUBA3C, U2AF1, U2AF2  WAS, WRN, WT1, XPO1, ZRSR2 |

Supplemental table 1. Next generation sequencing (NGS) panels per institution.
