# Supplementary material for: DNMT3A R882 Mutations Confer Unique Clinicopathologic Features in MDS Including a High Risk of AML Transformation
Source: Front Oncol. 2022 Feb 28;12:849376. doi: 10.3389/fonc.2022.849376 (PMC8918526; doi:10.3389/fonc.2022.849376)
Supplement: Supplementary file 5 [file Table_2.docx]

Supplemental table 2. Commonly mutated genes in MDS and AML

| Mutation Class | Mutation |
| --- | --- |
| Transcription | TP53 |
|  | RUNX1 |
|  | NPM1 |
|  | BCOR |
|  | ETV6 |
|  | PHF6 |
|  | GATA2 |
|  | CEBPA |
|  | WT1 |
| Splicing | SF3B1 |
|  | SRSF2 |
|  | ZRSR2 |
|  | U2AF1 |
| DNA Methylation | TET2 |
|  | IDH1 |
|  | IDH2 |
| Chromatin Modification | ASXL1 |
|  | EZH2 |
|  | SETBP1 |
|  |  |
| Receptors/Kinases | JAK2 |
|  | MPL |
|  | KIT |
|  | FLT3 |
| Cohesion | STAG2 |
|  | SMC1A |
| RAS Pathways | KRAS |
|  | NRAS |
|  | PTPN11 |
|  | CBL |
| Others | RAD21 |
|  | CSF3R |
|  | CALR |

MDS: myelodysplastic syndrome. AML: acute myeloid leukemia.
